# Early Diagnosis of WEBINO (Wall-Eyed Bilateral Internuclear Ophthalmoplegia) Syndrome Using Thin-Slice Gapless Diffusion-Weighted Imaging in an Acute Ischemic Stroke Patient: A Case Report

**DOI:** 10.7759/cureus.73372

**Published:** 2024-11-10

**Authors:** Hironori Mizutani, Keiichi Nakahara, Mitsuharu Ueda

**Affiliations:** 1 Neurology, Kumamoto University, Kumamoto, JPN

**Keywords:** brain infarction, diplopia, dwi, mri, webino syndrome

## Abstract

We report a case of acute ischemic stroke presenting as wall-eyed bilateral internuclear ophthalmoplegia (WEBINO) syndrome. A 71-year-old woman experienced transient diplopia, followed by the sudden onset of binocular misalignment, gait instability, and nausea. Neurological examination demonstrated exotropia and bilateral adduction impairment, consistent with WEBINO syndrome. An initial routine brain MRI did not reveal significant findings. However, thin-slice, gapless diffusion-weighted imaging (DWI) detected a high signal in the dorsal midline of the pons, confirming the diagnosis of acute pontine infarction. The patient received antiplatelet therapy, leading to a gradual improvement in her symptoms. This case highlights the need to consider WEBINO syndrome in cases of sudden-onset exotropia. It underscores the value of thin-slice, gapless DWI in identifying small brainstem infarcts that may be missed by routine imaging. Early and accurate diagnosis is essential for effective management and prognosis in such cases.

## Introduction

Wall-eyed bilateral internuclear ophthalmoplegia (WEBINO) syndrome is characterized by alternating exotropia and bilateral internuclear ophthalmoplegia, typically resulting from lesions in the pontine tegmentum or midbrain [1]. Stroke [2,3], multiple sclerosis [4], and other undetermined causes have been reported, though such cases are rare. The usefulness of diffusion-weighted imaging (DWI) MRI in detecting acute stroke has been well established [5]. The incidence of false-negative results has been reported to be higher in brainstem regions, particularly in the vertebrobasilar territory, compared with the carotid territory [6]. We report a case of acute brainstem infarction presenting as WEBINO syndrome, which was diagnosed early using thin-slice, gapless DWI.

## Case presentation

 A 71-year-old woman experienced a transient episode of diplopia lasting approximately five minutes, nine days prior to her hospital visit. During this episode, her left eye movement was restricted. The day before her visit, she experienced a sudden loss of focus and fell, accompanied by nausea and gait unsteadiness, which prompted her to seek medical care. Although she was a non-smoker, her medical history included hypertension and dyslipidemia.

**Figure 1:**
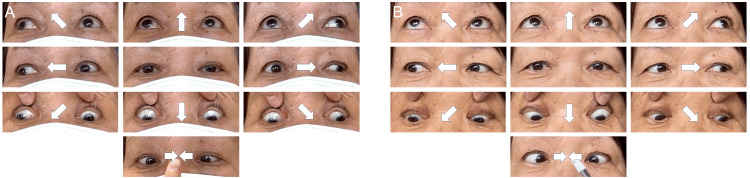
Ocular motility On admission (A), in the primary gaze, the patient shows alternating exotropia. On horizontal gaze, the abducting eye deviates fully, but the adducting eye does not cross the midline. Downgaze and upgaze are intact. Convergence is normal. At discharge (B), the adducting eye deviation improved.

Neurological examination revealed exotropia and bilateral impairment of adduction (Figure 1A). Convergence was preserved, and the oculocephalic reflex (OCR) was negative. The National Institutes of Health Stroke Scale (NIHSS) score was 1, indicating partial gaze palsy. Blood examination revealed elevated blood glucose (136 mg/dl) (reference range, 73-109 mg/dl), glycated hemoglobin (HbA1c) (6.5%) (reference range, 4.9-6.0%), low-density lipoprotein (LDL) cholesterol (266 mg/dl) (reference range, 65-163 mg/dl), and D-dimer (3.5 μg/ml) (reference range, <1.0 μg/ml), whereas other blood counts and biochemical markers were within normal limits. No atrial fibrillation was detected on ECG.

**Figure 2:**
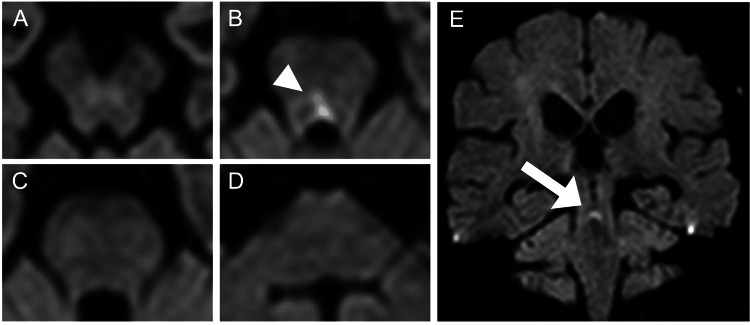
Brain MRI Axial (A: Midbrain, B: Upper pons, C: Middle pons, D: Lower pons) and coronal (E) diffusion-weighted image showing acute pontine tegmental infarction.

Initial routine brain MRI revealed no significant findings; however, additional thin-slice, gapless coronal DWI demonstrated a high signal on DWI and a low signal on apparent diffusion coefficient (ADC) in the dorsal midline of the superior pons (Figure 2). Magnetic resonance angiography (MRA) showed no significant stenosis, including in the basilar artery. The patient was diagnosed with acute ischemic stroke and was started on treatment with two antiplatelet agents. Further diagnostic assessments, including echocardiography, Holter monitoring, and carotid ultrasonography, revealed no significant abnormalities. Therefore, aspirin was prescribed for secondary prevention. The patient's symptoms, including exotropia and bilateral adduction impairment, were diagnosed as WEBINO syndrome. The negative OCR suggested a subnuclear oculomotor disorder. By hospital day 16, the exotropia and bilateral adduction impairment had improved (Figure 1B), and the patient was discharged home.

## Discussion

This case was an acute ischemic stroke presenting with WEBINO syndrome. Although a routine brain MRI on admission revealed no pontine or midbrain lesions, the exotropia and bilateral adduction impairment were indicative of WEBINO syndrome, prompting the use of thin-slice, gapless coronal DWI. This imaging revealed a high signal area in the dorsal midline of the pons. The pathophysiology of WEBINO syndrome involves the impairment of structures such as the paramedian pontine reticular formation (PPRF), the abducens nucleus, the vestibular nucleus, and the medial longitudinal fasciculus (MLF) [1], although many aspects of the mechanism remain unclear, WEBINO syndrome is usually associated with a convergence deficit, which was preserved in this case. Convergence involves the oculomotor nuclei of the midbrain but not the MLF [7]. Conversely, bilateral MLF disorders may be associated with bilateral internuclear ophthalmoplegia without exotropia.

A recent study suggests that there is a small group of neurons in the medial rectus subnucleus within the MLF, and dysfunction in this area may contribute to exotropia [8]. In two other previous case reports of WEBINO syndrome with preserved convergence, the lesion was small and located in the midbrain and superior pontine paramedian tegmentum [9,10]. These previous cases are valuable for understanding the MLF and surrounding anatomical structures. Multiple sclerosis and brainstem infarction are common causes of WEBINO [11], and it is important to suspect cerebral infarction, especially if there are risk factors for acute onset stroke. 

In the detection of acute cerebral infarction using DWI, high signal intensity in the brainstem often appears later than in cerebral cortical lesions [6], suggesting a higher false-negative rate in brainstem infarction compared to cerebral infarction. In this case, although routine DWI failed to detect the lesion, thin-slice gapless coronal DWI succeeded, demonstrating the utility of this method in reducing false negatives in the diagnosis of brainstem infarcts. Brainstem infarcts are generally smaller than cerebral infarcts, and lesions may be missed with conventional DWI that includes slice gaps. Gapless DWI addresses this issue, improving the detection rate of brainstem infarcts. In acute-onset WEBINO syndrome, brainstem thin-slice gapless coronal DWI should be considered, as brainstem infarction is highly suspected. 

In clinical practice, it is not uncommon to encounter patients with ocular symptoms due to stroke, and accurate recognition of these symptoms is essential for the localization of the lesion. When sudden-onset bilateral exotropia is observed, WEBINO syndrome should be considered, and the possibility of stroke must be investigated and treated promptly.

## Conclusions

This case underscores the utility of thin-slice gapless DWI in the early detection of brainstem infarcts that may not be visible on conventional brain MRI. Although the initial MRI showed no abnormalities, the use of gapless DWI successfully identified an acute pontine infarction. This case emphasizes the importance of considering WEBINO syndrome in cases of sudden-onset exotropia and illustrates the value of thin-slice gapless DWI in identifying small brainstem lesions that are often missed with routine imaging techniques.
